# Modeling the Potential for Vaccination to Diminish the Burden of Invasive Non-typhoidal *Salmonella* Disease in Young Children in Mali, West Africa

**DOI:** 10.1371/journal.pntd.0005283

**Published:** 2017-02-09

**Authors:** Kristin Bornstein, Laura Hungerford, David Hartley, John D. Sorkin, Milagritos D. Tapia, Samba O. Sow, Uma Onwuchekwa, Raphael Simon, Sharon M. Tennant, Myron M. Levine

**Affiliations:** 1 Center for Vaccine Development and Institute for Global Health, University of Maryland School of Medicine, Baltimore, MD, United States of America; 2 Department of Epidemiology & Public Health, University of Maryland School of Medicine, Baltimore, MD, United States of America; 3 James M. Anderson Center for Health Systems Excellence, Cincinnati Children’s Hospital Medical Center, Cincinnati, OH, United States of America; 4 Department of Medicine, University of Maryland School of Medicine, Baltimore, MD, United States of America; 5 Baltimore VA Medical Center GRECC (Geriatric Research, Education, and Clinical Center), Baltimore Maryland; 6 Department of Pediatrics, University of Maryland School of Medicine, Baltimore, MD, United States of America; 7 Centre pour le Développement des Vaccins, Mali (CVD-Mali), Bamako, Mali, Africa; Johns Hopkins Bloomberg School of Public Health, UNITED STATES

## Abstract

**Background:**

In sub-Saharan Africa, systematic surveillance of young children with suspected invasive bacterial disease (e.g., septicemia, meningitis) has revealed non-typhoidal *Salmonella* (NTS) to be a major pathogen exhibiting high case fatality (~20%). Where infant vaccination against *Haemophilus influenzae* type b (Hib) and *Streptococcus pneumoniae* has been introduced to prevent invasive disease caused by these pathogens, as in Bamako, Mali, their burden has decreased markedly. In parallel, NTS has become the predominant invasive bacterial pathogen in children aged <5 years. While NTS is believed to be acquired orally via contaminated food/water, epidemiologic studies have failed to identify the reservoir of infection or vehicles of transmission. This has precluded targeting food chain interventions to diminish disease transmission but conversely has fostered the development of vaccines to prevent invasive NTS (iNTS) disease. We developed a mathematical model to estimate the potential impact of NTS vaccination programs in Bamako.

**Methodology/Principal Findings:**

A Markov chain transmission model was developed utilizing age-specific Bamako demographic data and hospital surveillance data for iNTS disease in children aged <5 years and assuming vaccine coverage and efficacy similar to the existing, successfully implemented, Hib vaccine. Annual iNTS hospitalizations and deaths in children <5 years, with and without a *Salmonella* Enteritidis/*Salmonella* Typhimurium vaccine, were the model’s outcomes of interest. Per the model, high coverage/high efficacy iNTS vaccination programs would drastically diminish iNTS disease except among infants age <8 weeks.

**Conclusions/Significance:**

The public health impact of NTS vaccination shifts as disease burden, vaccine coverage, and serovar distribution vary. Our model shows that implementing an iNTS vaccine through an analogous strategy to the Hib vaccination program in Bamako would markedly reduce cases and deaths due to iNTS among the pediatric population. The model can be adjusted for use elsewhere in Africa where NTS epidemiologic patterns, serovar prevalence, and immunization schedules differ from Bamako.

## Author Summary

A surveillance program at Gabriel Touré Hospital in Mali observed a high burden of invasive disease caused by non-typhoidal *Salmonella* (iNTS). This surveillance program was originally instituted to measure the amount of invasive disease (e.g., septicemia, meningitis) caused by two bacteria that invade the respiratory tract: *Haemophilus influenzae* type b (Hib) and Streptococcus pneumoniae (pneumococcus). While documenting the burden of these pathogens, the surveillance program also found that serotypes of iNTS, mainly *Salmonella* Typhimurium and *Salmonella* Enteritidis, were common causes of severe invasive disease. As the number of cases of Hib and pneumococcus markedly decreased following the introduction of relevant vaccines, the relative threat of iNTS increased. Little is known about the reservoir of iNTS, whether it resides in humans, animals, or the environment, or how it is spread to susceptible children. Without this knowledge, it is not possible to employ certain disease control methods useful in interrupting the transmission of other pathogens. Therefore, vaccination remains the one promising control strategy for this disease. Our research modeled the potential effects of introducing an iNTS vaccine. The findings are of great importance to Mali and other developing countries where young children are at a high risk of developing iNTS disease.

## Introduction

In industrialized countries, non-typhoidal *Salmonella* (NTS) predominately causes gastroenteritis [1, 2]. However, in sub-Saharan Africa the NTS serovars *S*. Typhimurium and *S*. Enteritidis have become recognized as important causes of severe invasive bacterial disease (e.g., septicemia, meningitis, bacteremia) with high case fatality rates [2, 3, 4]. Infants age 6–11 months and toddlers age 12–23 months exhibit the highest incidence of severe invasive NTS (iNTS) disease [5]. Whereas host factors such as malnutrition and co-infection with malaria and HIV may contribute to the higher burden of iNTS disease (i.e., high case fatality rate and prevalent cause of bacteremia) in this region compared to industrialized countries [6–7], fundamental differences in the circulating NTS strains from sub-Saharan Africa are also evident. Available evidence suggests that the vast majority of the *S*. Typhimurium strains from cases of iNTS disease in sub-Saharan Africa are multi-locus sequence type 313 (ST313), a genotype unique to Africa that has undergone extensive genomic degradation [8–9]. As the burdens of invasive disease due to *Haemophilus influenzae* type b (Hib) and *Streptococcus pneumoniae* have plummeted in recent years in sub-Saharan Africa following the introduction of Hib conjugate and multivalent pneumococcal conjugate vaccines [10–11], recognition of the need to address iNTS disease has increased [12]. Lack of information on the reservoirs and vehicles of transmission of iNTS in sub-Saharan Africa limits opportunities to utilize classic epidemiologic interventions to control iNTS disease. However, successful vaccination programs implemented to control other invasive diseases prevalent among pediatric populations in Mali and other countries of sub-Saharan Africa have stimulated interest in the development of vaccines to control iNTS disease.

Several candidate vaccines under development have shown promise in protecting against invasive *S*. Typhimurium and *S*. Enteritidis disease in animal models [13, 14]. These include a bivalent conjugate vaccine based on covalently linking the core and O-antigen polysaccharides of *S*. Typhimurium (a Group B [O:4] serovar) and *S*. Enteritidis (a Group D [O:9] serovar) to the respective Phase 1 flagellin subunits (FliC) of each of these serovars [14–16], a live attenuated oral vaccine [17–18], and a bivalent Generalized Modules for Membrane Antigens (GMMA) vaccine consisting of outer membrane protein blebs from *S*. Typhimurium and *S*. Enteritidis that include lipopolysaccharide [12, 14]. These vaccines also have the potential to provide cross protection against other NTS serovars within *Salmonella* O Group B (e.g., *S*. Stanleyville) and O Group D (e.g., *S*. Dublin) [13, 16, 18]. Our research modeled the decrease in the number of cases and deaths attributable to iNTS in children < 5 years of age following the programmatic introduction of a NTS vaccine utilizing the same Expanded Program on Immunization (EPI) infrastructure that successfully delivered Hib and pneumococcal conjugate vaccines and that drastically reduced the number of cases of invasive disease caused by those pathogens.

## Methods

### Data sources

Census data from the National Institute of Statistics (INSTAT) of Mali from 2009 [19] provided the pediatric population of different age groups of interest in Bamako as denominators for the model. Crude birth rate and age-specific all-cause mortality data for specific pediatric age groups in Bamako came from Demographic and Health Surveys (DHS) of 2001, 2006 and 2012 [20–22] (Table 1). The highest and lowest rates reported across the years of DHS reports were used to establish a range of probable values with the intermediate of the three values used as the initial parameter value.

**Table 1:** Background Birth and Mortality Rates* from DHS, Bamako [20–22].

Rates	Variables	Parameter Value (Range)
Birth Rate	*ν*	0.039 (0.038, 0.042)
All-cause Mortality Rate	μ[a]	
Neonatal (<1 mo)	*a* = 1	0.035 (0.028, 0.056)
Post-neonatal (1 mo—<12 mo)	*a* = 2–9	0.031 (0.014, 0.038)
Child (12–59 mo)	*a* = 10	0.045 (0.018, 0.044)

* Per 10,000 individuals of that age-group in the population

The burden of invasive bacterial disease caused by NTS in Mali was first identified during systematic surveillance of the incidence of bacterial pathogens begun in 2002 at l’Hôpital Gabriel Touré (HGT), Bamako, Mali. The surveillance program established by the Center for Vaccine Development, Mali (CVD-Mali) and the Center for Vaccine Development (CVD), University of Maryland School of Medicine, was designed to identify bacterial pathogens associated with invasive disease among consented enrolled patients <15 years of age admitted to HGT with fever or clinical signs of invasive bacterial disease [5]. This hospital-based surveillance was conducted under a protocol approved by the Ethics Committee of the Faculté de Médecine, Pharmacie et Odontostomatologie in Bamako, Mali and the University of Maryland Institutional Review Board. Consent was documented on a written form. If the participant's parent or guardian was illiterate, they listened to an audiotaped version of the consent form in their local language and questions were so answered in the presence of a witness.

**Fig 1:**
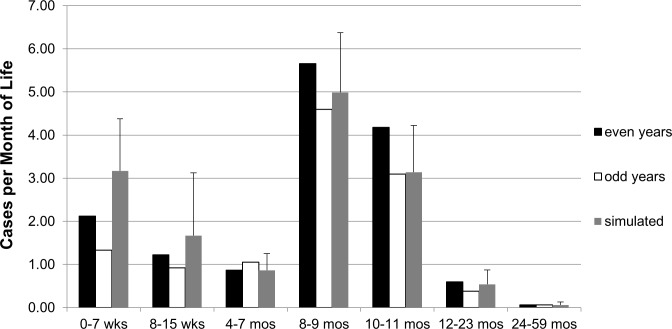
Average Number of Cases per Month by Age Group Occurring in Even and Odd Years of Surveillance and in Model Simulations.

We used anonymized data on 515 pediatric patients under five years of age who were admitted to HGT with laboratory-confirmed iNTS disease between July 1, 2002 and June 30, 2014 to develop and validate the model parameters. Numbers of cases within specific age groups, pooled across even years of the HGT surveillance (i.e., 2002, 2004, 2006, 2008, 2010, 2012), were used with denominators from INSTAT [19] to generate the age group-specific hospitalization rates of severe iNTS disease (Table 2). Case fatality rates for the model were fatal cases divided by total cases per age group (Table 3). The model was then validated by comparing the number of cases and deaths due to iNTS per year estimated by the model, without accounting for vaccination effects, against the data from the odd years of HGT surveillance. Data were pooled as means across the years of surveillance because the number of cases per age-group per year was small (Fig 1). Multiple years of data were included to provide more robust estimates for the Malian pediatric population and to encapsulate some of the variability over time.

**Table 2:** Hospitalization Rates* from Invasive NTS based on 2002–2012 Serovar Distribution from Even Years of HGT Surveillance.

Age Range	Variable (β[*a*])	Parameter Value (95% CI)
0–7 weeks	*a* = 1–3	0.41 (0.13, 0.69)
8–15 weeks	*a* = 4–5	0.24 (0.01, 0.47)
4–8 months	*a* = 6	0.17 (0.06, 0.27)
9 months	*a* = 7	1.08 (0.37, 1.71)
10–11 months	*a* = 8	0.80 (0.16, 1.45)
12–23 months	*a* = 9	0.11 (0.08, 0.14)
24–59 months	*a* = 10	0.01 (0.01, 0.10)

* Per 10,000 individuals of that age-group in the population from National Institute of Statistics of Mali [19]

**Table 3:** iNTS Case Fatality Rates from Even Years of HGT Surveillance (η[a]).

Serovar Distribution	0–7 weeks of age (*a* = 1–3) Parameter Value (95% CI)	8+ weeks of age (*a* = 4–10) Parameter Value (95% CI)
2002–2012 observed serovar distributions	0.75 (0.41, 0.93)	0.15 (0.09, 0.22)
*S*. Typhimurium only	0.0 (0.0, 0.85)	0.13 (0.06, 0.23)
*S*. Enteritidis only	0.86 (0.42, 0.99)	0.18, (0.09, 0.31)

The proportion of hospitalizations with *S*. Typhimurium and *S*. Enteritidis serovars has been seen to change over time. In particular, from 2008 to the present, the incidence of invasive *S*. Typhimurium infections has decreased, while the incidence of invasive *S*. Enteritidis infections has increased [5]. Moreover, these serovars exhibit different case fatality rates. Therefore, serovar-specific case fatality rates for hospitalized children were also calculated based on the HGT surveillance data.

The expected coverage for an iNTS vaccine was estimated based on data from Hib vaccine implementation in Bamako. Vaccination coverage estimates came from an immunization coverage survey undertaken in 2015 among a sample of infants 6–8 months of age in the population as part of prospective demographic surveillance in the Djikoroni-para quartier of Bamako. The demographic surveillance system allowed population-based estimates to be derived as was done for the Global Enteric Multicenter Study (GEMS) [23–24]. Sixty-one mothers or other caretakers of infants 6–8 months of age were asked if they had an immunization card and 60 were able to show the card. The narrow infant age range was selected to document not only evidence of receipt of Hib vaccine but to provide information on the timeliness of immunization which is important to the success of Hib and NTS vaccination as a public health tool. Among these 61 Djikoroni-para infants, 60 had received at least one dose of Hib vaccine 60/61 (98.4%) and 55 had received all three doses of Hib vaccine (55/61, 90.2% full coverage). This Hib vaccine coverage information from Bamako was used as the starting point for our simulations, since it was drawn directly from our modeled population. Hib coverage data from Kenya [26] was utilized to generate wider intervals of coverage values from a larger study sample and broader population data. Coverage data for alternative vaccination programs and booster vaccinations were based on the measles vaccine program implemented in Mali, which targeted children of the same scheduled ages as proposed in the model [33].

Assumptions on the expected efficacy of the vaccines under development to prevent iNTS disease in Mali were based on assessments of the efficacy of Hib conjugate in a randomized clinical trial in The Gambia [24] and from post-licensure impact evaluations on Hib disease in Mali [10], Kenya [26] and Uganda [29] and a 9-valent pneumococcal conjugate vaccine efficacy trial in The Gambia [30]. Hib conjugate was highly effective in diminishing the disease burden when administered routinely through the Expanded Program on Immunization in Mali [10], Kenya [26] and other African countries [29]. Efficacy for booster vaccination doses and a catch-up campaign program were based on anticipated results similar to those exhibited by the Hib catch up campaign and booster vaccine interventions performed in the United Kingdom [34–35]. In certain populations, such as those with a high prevalence of HIV cases, immune suppression decreases the amount of protection granted by vaccination against Hib [25]. While the pediatric population of Bamako does not exhibit high levels of HIV, a scenario with low vaccine efficacy due to immune suppression such as seen by Madhi et al., in South Africa (a 20% decrease in each vaccine efficacy parameter) was modeled.

The invasive disease such as meningitis, septicemia, bacteremia and septic arthritis caused by invasive non-typhoidal *Salmonella* is clinically indistinguishable from those types of clinical infections caused by Hib. Each of these pathogens traverses a mucosal barrier leading to a bacteremia during which the bacterial pathogens are cleared by fixed macrophages residing in organs of the reticuloendothelial system. In the case of Hib, it is upper respiratory mucosa that is traversed, while for iNTS it is believed to be intestinal mucosa. Bacteremic organisms that reach the meninges, synovia and pleura can cause meningitis, septic arthritis and empyema, respectively. The NTS conjugate vaccines under development elicit serum antibodies that exhibit both bactericidal and opsonophagocytic functional properties [31–32, 36], like the antibodies stimulated by Hib conjugate vaccines [25, 37–40]. Thus, there exist striking pathogenetic, clinical and epidemiologic similarities between iNTS and Hib pathogens and similar functional activities are exhibited by the antibodies stimulated by the parenteral NTS conjugate vaccines (in animals) and by Hib conjugate vaccine in human infants. Therefore, we assumed a similar efficacy and coverage for the NTS vaccine as was observed with Hib conjugate vaccine in the infant and toddler population in Bamako (and elsewhere in sub-Saharan Africa). Relying on Hib vaccination efficacy data allowed us to validate the iNTS vaccine implementation within the model and allowed for reliable comparisons of the protection potentially granted by the iNTS vaccine and various immunization schedules.

### Model description

**Fig 2:**
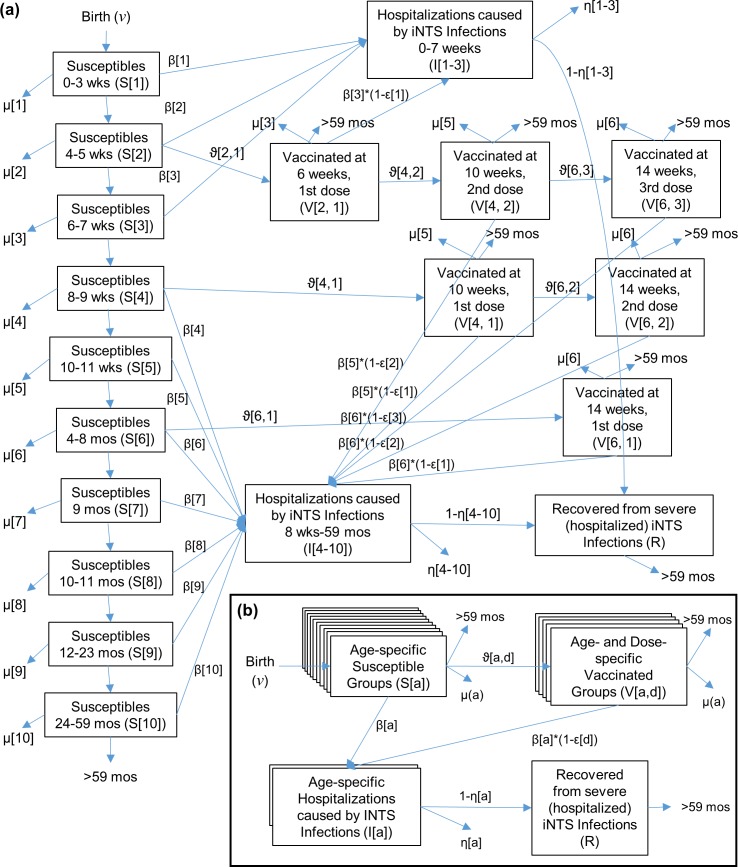
Diagram of the Model. (a) Base vaccination model, with vaccinations at 6, 10, and 14 weeks of life, (b) Generalized diagram of the model for alternative vaccination schedules.

The incidence and epidemiologic features of iNTS infections among young children sufficiently severe to result in hospitalization were captured using an age-structured Markov chain infectious disease model including Susceptible, Infected, and Recovered status groups. A diagram of the model (Fig 2A) with vaccine administered at 6, 10, and 14 weeks of life, as was used in the Hib vaccination initiative, was used as the baseline for developing a generalized model capturing all modeled vaccination schedules as illustrated in Fig 2B.

Each age group (*a*) included an age-specific number of children susceptible to (S[*a*], 10 groups, where *a =* 1–10), hospitalized with (I[*a*], 4 groups, where *a =* 1–3, 4–8, 9, 10), or vaccinated by dose (*d*) against (V[*a*,*d*], where *a* varied for different scenarios and *d =* 1–5) severe iNTS disease. Children who recovered (R) from severe hospitalization were considered as a single group. Age categories were established by examining statistically significant variation in incidence and case fatality rates of iNTS (p<0.05) within the HGT surveillance data, and ages at which the EPI vaccinations were scheduled.

Neonates entered the model based on the population birth rate reported for Bamako (*ν*, Table 1) and were considered to be hospitalized with iNTS disease at the same incidence as other infants < 2 months of age (Table 2). Children at any age were subject to the age-specific all-cause mortality rates (μ[*a*], Table 1) reported in the DHS. Susceptible children (S[*a*]) were moved to a hospitalized status (I[*a*]) at age-specific iNTS hospitalization rates (β[*a*], Table 2). Children hospitalized with iNTS disease experienced mortality at age-specific case fatality rates (η[*a*], Table 2) or moved to a recovered status (R). The length of iNTS infection was constrained to a single two-week time step corresponding to the observed duration of iNTS clinical disease in hospitalized Bamako children. Any susceptible children who did not suffer hospitalized iNTS disease, iNTS fatality, or all-cause mortality graduated into the next susceptible age group at a rate appropriate to the two-week time step of the model. All surviving children exited the model upon reaching five years of age, when the rate of hospitalizations due to iNTS rapidly declines [5].

Vaccination against iNTS was initially modeled as a program which occurred at ~6, ~10, and ~14 (*a* = 2,4,6, respectively) weeks of life to match the same three-dose infant immunization schedule as Hib conjugate. Children potentially received one, two, or three (dose *d* = 1–3, respectively) doses of the vaccine with age-specific and dose-specific coverage rates (ϑ[*a*,*d*]). For vaccinated children, the rate of hospitalization due to iNTS disease was applied only to the proportion of children without an effective vaccination (1-ε[*d*]). Vaccine protection was assumed to persist through early childhood, so successfully immunized children remained protected until they aged out of the model.

Our major outcome measures were the number of cases and deaths due to iNTS disease. To generate an overall distribution of potential values describing the natural epidemiologic behavior of the disease without implementation of a vaccination program, 1000 simulations were run with key model variables (i.e., birth rate, incidence of hospitalization of iNTS cases, case fatality, and background all-cause mortality rates) randomly drawn each time from a uniform distribution based on the limits of the probable range around each parameter. After being used to simulate the ‘average’ dynamics in absence of vaccine use, a second set of 1000 simulations was used to assess the effect of different assumptions about vaccine efficacy and coverage in the model. Key variables were sampled as before and values for vaccine efficacy and coverage drawn from triangular distributions based on each parameter value and its associated probable range under different scenarios. The influence of each parameter on the number of iNTS cases and fatal iNTS cases generated by the model was assessed by sampling each parameter individually, while holding all other parameter values constant. The ranges of iNTS cases and fatal iNTS cases generated by these simulations were summarized in tornado plots. Next, specific effects of vaccination were examined in a birth cohort of 75,978 children, corresponding to the annual births for Bamako. The expected number of cases and number of fatal cases in children < 36 months of age within this cohort was generated under unvaccinated conditions, vaccinated conditions with 100% coverage and efficacy implemented at 6 weeks of life, and vaccinated conditions with coverage and efficacy matching the model parameters described in Table 4 at 6, 10, and 14 weeks of life. Additionally, the effects of high, mid, and low vaccine efficacy levels on the overall number of cases and case fatalities were examined by dose, based on the ranges around these parameters.

**Table 4:** NTS Vaccination Characteristics.

Dose	Coverage (ϑ[*a*,*d*]) Parameter Values (Probable Range)	Efficacy (ε[*d*]) Parameter Values (Probable Range)
One dose (*d* = 1)^*^	0.98 (0.89, 0.98)	0.45 (0, 0.82)
Two doses (*d* = 2)^*^	0.93 (0.83, 0.96)	0.90 (0.68, 0.97)
Three doses (*d* = 3)^*^	0.90 (0.78, 0.90)	0.95 (0.83, 0.98)
Booster (*d* = 4)^**^	0.88	0.85
Catch-up campaign (*d* = 5)^***^	-	0.6–0.8

* Coverage based on Bamako and Kenya Hib conjugate Field Data [10, 24, 26]; Efficacy based on Gambia Hib conjugate trial data [24]

** Coverage based on Mali MCV1 vaccine coverage, 2004 [33]; Efficacy based on an anticipated 10% decrease

*** Anticipated range simulated

To assess the effects of serovar- specific severity, the vaccination program was modeled using case fatality rates representing only *S*. Typhimurium or only *S*. Enteritidis as causal agents (Table 3). Since the overall incidence of iNTS disease has not changed significantly with the documented serovar shift, we maintained the same overall incidence rates regardless of underlying causal agent. Another 1000 simulations were performed to assess effects of varying the parameter values across the probable ranges for case fatality rates of these two serovars on the number of severe iNTS cases and fatal iNTS cases.

The same approach was used to simulate effects of alternative three-dose EPI vaccination regimens. For example, the first two doses were administered at 6 (*a =* 2) and 10 (*a =* 4) weeks or at 10 (*a =* 4) and 14 (*a =* 6) weeks of life (concomitant with two doses of pentavalent and pneumococcal conjugate vaccine) and the third NTS vaccine dose was administered as a booster (dose *d* = 4) at either age 9 months (*a =* 7) (with measles containing vaccine dose 1 [MCV1]) or at 12 or 15 (*a =* 9) months of age concomitant with MCV2. We also modeled a rapid mass immunization catch-up campaign (dose *d* = 5) targeting all children of age 6–23 (*a =* 6–9), 9–23 (*a =* 7–9), or 12–23 (*a =* 9) months of age concomitant with the onset of adding iNTS vaccination to the routine young infant EPI. In each of these schedules, the precise ages at time of vaccination, although ideally targeted at specific weeks of life, in fact vary and are often delayed by several weeks in Bamako. To allow for this, the number of children admitted to the HGT who received one, two, or three doses of vaccine any time within the relevant month of life was used to calculate the sample mean coverage levels among hospital admissions, and not by specific week of implementation. These coverage levels fell within the confidence intervals of the Kenyan coverage data reported by Cowgill et al. [26] that were used to parameterize the model.

Model development and analyses were performed using R version 3.0.1 and utilizing the Markovchain package, version 0.5 [27] for the development of the model and the Triangle package, version 0.1 [28] for vaccine efficacy and vaccine coverage variable distribution analysis. The code developed for the model and the case data used to develop the model parameters are available on a public GitHub repository [41].

## Results

### Modeling age-related iNTS disease burden in the absence of vaccination

Based on the parameter values determined from even years, our model predicted a similar number of hospitalized iNTS disease cases for most age groups as was observed during the odd years of HGT surveillance data (Fig 1), with 37 cases per year, including 7 fatal cases, occurring in a non-vaccinated population. The 1000 model runs generated a range of 14–64 cases per year (with interquartiles of 29 and 39) and a range of 2–14 fatal cases per year (with interquartiles of 5 and 8), sampling from the probable range of model parameters. Hospital surveillance records did not include neonatal cases who died of iNTS infection before leaving the hospital after birth, so our model assumed a similar level of incidence among this youngest age group as was observed for other children less than one month old. This assumption generated a simulated overestimation of cases in the youngest age group compared to the observed, but attempted to include neonatal cases and iNTS related deaths in our outcome measurements. Varying the parameter estimates used for hospitalized infection rate, all-cause mortality rate, and case fatality rate across the range of each parameter led to a mean of 34 cases per year (ranging from 14–64, with interquartiles of 29 and 39) and 7 fatal cases per year (ranging from 2–14, with interquartiles of 5 and 8), based on 1000 runs of the model.

### Modeling the effects of iNTS vaccination

**Fig 3:**
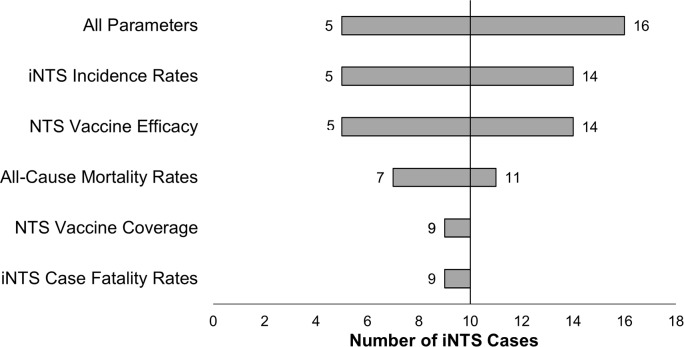
Changes in the Number of Hospitalized iNTS Cases across the Probable Ranges of Model Parameters.

**Fig 4:**
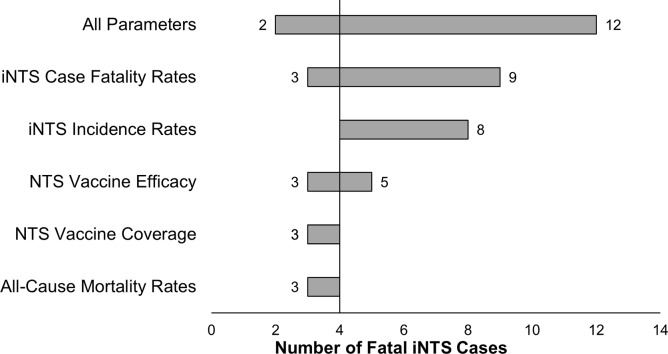
Changes in the Number of Fatal iNTS Cases across the Probable Ranges of Model Parameters.

**Fig 5:**
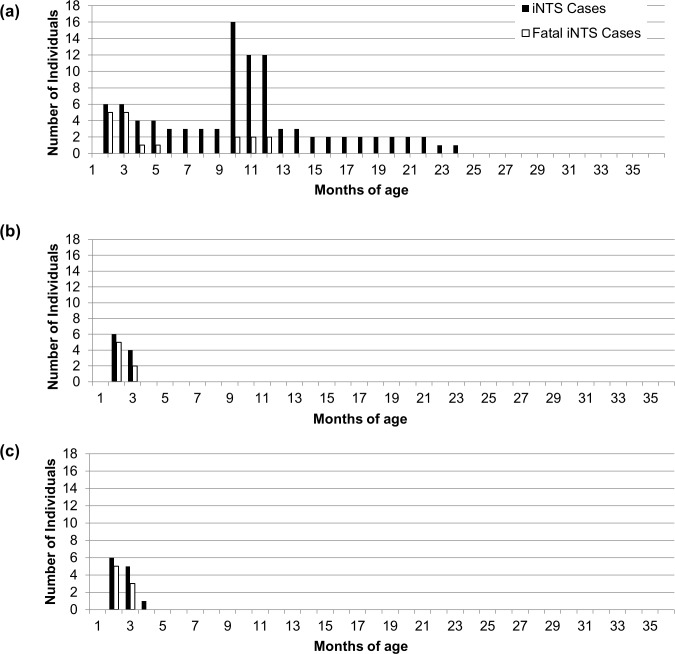
Total Cases and Fatal Cases due to iNTS Disease within a Birth Cohort over Time. (a) in unvaccinated conditions, (b) with a vaccine administered at 6 weeks of life with 100% coverage and 100% efficacy, and (c) with vaccines administered at 6, 10, and 14 weeks of life with vaccine coverage and efficacy as described in Table 4.

One thousand model runs with varying parameter estimates for birth rate, incidence of hospitalization of iNTS cases, case fatality, background all-cause mortality rates, vaccine efficacy and coverage with three doses of vaccine administered at 6, 10, and 14 weeks of life generated a mean of 9 cases per year (ranging from 5–16, with interquartiles of 11 and 6) and 3 fatal cases per year (ranging from 2–12, with interquartiles of 2 and 5). The greatest change in the number of iNTS cases occurred as the incidence rate was sampled across its probable range, while the least amount of change was generated by sampling across vaccine coverage (Fig 3). The greatest change in the number of fatal iNTS cases was driven by the case fatality rate (Fig 4). In observing the total number of cases and the number of deaths due to iNTS among a birth cohort, as presented in Fig 5, the vaccination parameters of the model functioned as expected. If a NTS vaccine was implemented with 100% coverage and 100% efficacy, all cases following an initial dose of vaccine were prevented and all fatal cases were averted. Furthermore, when the model was run with parameters based on the Hib vaccination field trials and post-introduction impact assessments in Africa, the results were very similar to a vaccine with perfect coverage and efficacy. Almost all cases among the birth cohort were prevented even after a single dose, and all cases in the cohort were prevented after two doses.

Modeling vaccine coverage and efficacy for an iNTS vaccine equivalent to levels observed with Hib (with routine young infant immunization only and with no catch-up campaign), the number of hospitalized iNTS cases per year decreased by 73% (from 37 to 10 cases) and the number of deaths decreased by 43% (from 7 to 4 deaths). These estimates, based on the distribution of *S*. Enteritidis and *S*. Typhimurium cases that was observed during the 2002–2014 surveillance, reflect the effect of direct protection alone (i.e., with no adjustment for indirect protection from “herd immunity”) and without a catch-up campaign.

**Fig 6:**
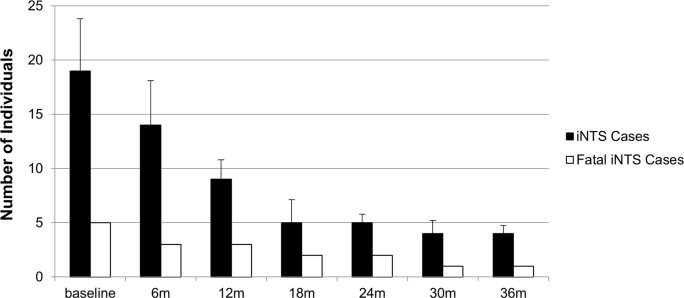
Number of Severe iNTS Cases and Deaths per Cumulative 6-Month Interval after Implementing a 3-dose Vaccine Program.

Effects of varying the parameter values for vaccine efficacy, number of doses, serovar distributions, and vaccination schedules are shown in Table 5. Even at the lowest ranges of vaccine efficacy, our model predicted a range of only 4–23 cases per year, based on 1000 simulation runs. This equates to prevention of more than half of the pediatric cases each year if the vaccine exhibits similar efficacy as might be seen with the Hib vaccine in immunodeficient populations. At higher levels of efficacy, as much as 78% of severe iNTS cases (29 cases/year) were averted. The highest level of protection was granted by a 3-dose vaccination schedule targeting infants at 6, 10, and 14 weeks of life, which resulted in a 73% decrease in the annual number of severe iNTS cases with moderate vaccine efficacy levels. This varied from 21–29 cases prevented and 2–3 child lives saved per year among the pediatric population of Bamako, based on the vaccine efficacy levels observed in the Hib conjugate field trial in The Gambia [24] and the post-implementation effectiveness assessment in Mali [10]. The increased immunity over time among the population was captured through the number of hospitalized iNTS cases and deaths per 6-month intervals after vaccine implementation, compared to a 6-month pre-vaccination baseline interval, as illustrated in Fig 6.

**Table 5:** Number of Cases Prevented and Deaths Averted per Year^+^ Depending on Vaccine Efficacy, Serovar Distribution, and Vaccination Schedule.

Vaccination scenarios	Cases Prevented (%)	Deaths Averted (%)
Three-dose^*^ vaccination	27 (73%)	3 (43%)
Two-dose vaccination with booster at age ~9 months	27 (73%)	3 (43%)
Three-dose^*^ vaccination with low efficacy due potential immune suppression	19 (51%)	2 (29%)
Low predicted vaccine efficacy	21 (57%)	2 (29%)
High predicted vaccine efficacy	29 (78%)	3 (43%)
*S*. Typhimurium as the only iNTS causal agent^**^	28 (74%)	4 (80%)
*S*. Enteritidis as the only iNTS causal agent^**^	27 (75%)	4 (44%)

^+ ^Numbers provided reflect the cases prevented and deaths averted per year once the vaccine has been routinely administered and reached a steady state throughout the pediatric population

* Doses administered at ~6, 10, and 14 weeks of life

** Compared to theoretical unvaccinated populations with single serovar distributions

**Fig 7:**
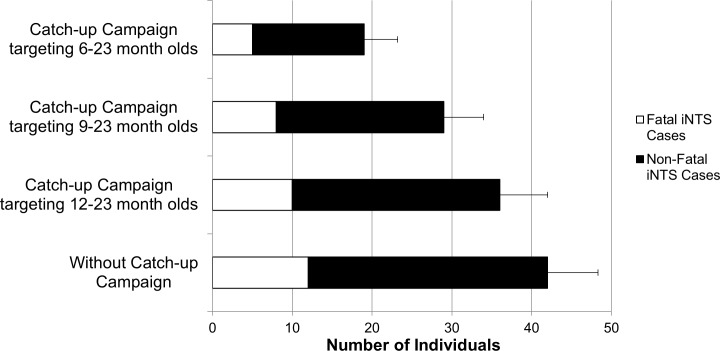
Number of Severe iNTS Cases and Deaths within the First 36 Months after Introduction of Routine NTS Vaccination and Various Catch-up Campaign Schedules.

If a catch-up vaccination campaign was implemented simultaneously along with the introduction of a three-dose young infant EPI vaccination strategy, protection occured sooner among older age children who remained at risk. Fig 7 illustrates the effects of such a catch-up campaign targeting various age groups. The addition of the catch-up campaign prevented at least four and as many as 12 additional cases of severe iNTS during the first three years following the catch-up campaign and start of the vaccine initiative compared to the three-dose schedule without the catch-up campaign.

Investigating the effects of different serovar distributions that have been observed over time, our model predicted that if *S*. Enteritidis, with its higher case fatality rate, was the only iNTS serovar causing disease, 44% of deaths per year would be averted through vaccination, with a range of 5–37 cases per year including 2–18 fatal cases, across 1000 simulation runs. If *S*. Typhimurium returned as the dominant serovar, up to 80% of iNTS deaths per year would be averted, with a range of 4–31 cases per year, including 1–8 fatal cases.

## Discussion

Implementation of programmatic use of the Hib conjugate vaccine among the pediatric population in Bamako, Mali was extremely successful and reduced the burden of invasive Hib disease hospitalizations by 83% among infants within three years of vaccine introduction [10]. Our results show that if we utilize the same EPI infrastructure to deliver a bivalent conjugate vaccine to prevent invasive disease due to *S*. Enteritidis and *S*. Typhimurium, it is reasonable to expect a comparable level of protection and reduction of iNTS cases as was seen with Hib vaccine. Our iNTS model captured the current burden of iNTS in the population and assessed potential effects of various vaccine implementation scenarios. Even alternative vaccination schedules with fewer doses of iNTS vaccine predicted a notable reduction in the burden of iNTS disease. Additionally, if a one-time catch-up campaign is implemented concomitantly with routine vaccination of young infants and if it targets the highest risk age group (children in the second half of the first year of life), our model predicted additional cases of severe iNTS disease deaths would be averted in the first years after introducing the vaccine. If field trials confirm the efficacy of the NTS vaccines currently under development, their future implementation within the EPI could markedly diminish the morbidity and mortality from one of the predominant remaining burdens of invasive bacterial disease among pediatric populations in sub-Saharan Africa. Since neither the reservoir of infection nor the modes of transmission of NTS to young children have heretofore been identified, vaccination currently represents the most plausible interventional strategy for reducing the burden of iNTS disease. Furthermore, the model we have developed could be applied to estimate the effects of implementing an iNTS vaccine in other regions of sub-Saharan Africa, providing the same integrity of information on age-specific case and fatality rates.

Two candidate NTS vaccines are progressing towards clinical trials. One candidate developed by the GSK Vaccines Institute of Global Health consists of a bivalent parenteral *Salmonella* Enteritidis/*S*. Typhimurium vaccine based on Generalized Modules for Membrane Antigens (GMMA) technology [14, 42–43]. The second NTS candidate, developed by the Center for Vaccine Development of the University of Maryland School of Medicine (CVD) and its industrial partner, Bharat Biotech, International (BBI) of Hyderabad, India, contains *S*. Enteritidis and *S*. Typhimurium conjugate vaccines consisting of the core plus O polysaccharide of those serovars covalently linked to Phase 1 flagellin subunits of the homologous serovar [13–16, 44].

As each vaccine moves towards clinical trials, a Target Product Profile (TPP) must be created that by necessity incorporates multiple assumptions and predictions that guide the development of the project for multiple years before clinical data become available to corroborate or refute the TPP assumptions. The TPP, which must be crafted early in the development of the candidate vaccine, provides a roadmap as it defines the type of vaccine, the route of administration, the target populations and sub-populations to be vaccinated, the number of doses to be administered and the intended immunization schedule for the target populations. The TPP also proposes limits for the expected reactogenicity (local and systemic), the level of efficacy to be achieved, the duration of protection, when a booster dose might be needed, the storage conditions, the vaccine formulation(s), the presentation of the vaccine, whether an adjuvant will be included and what preservative will be present in multi-dose vials. The design of the preclinical toxicology test, the formulations of vaccine to be tested, the design of the Phase 1 and 2 clinical trials, and of the ultimate pivotal Phase 3 efficacy trial all follow guidance provided by the TPP. The mathematical model described herein includes assumptions contained within one TPP. Even at early stages in development of the candidate vaccines to prevent iNTS disease, a mathematical model of what the vaccine might achieve at the future public health level becomes a useful, hopefully predictive, tool. Modifying the parameters of the model offers insights on what the vaccine can achieve.

Our model has been used to assess the impact of introducing a bivalent NTS vaccine on decreasing the number of hospitalized iNTS cases and fatalities caused by the two most prevalent iNTS serovars currently found in the Malian pediatric population, *S*. Enteritidis and *S*. Typhimurium. However, other serovars have been identified among a minority of hospitalized cases of iNTS that theoretically could also be prevented. Indeed the bivalent vaccines currently in development offer the prospect of cross protection against other serovars. The bivalent conjugate vaccine described by Simon et al. [16], for example, may offer such cross protection by targeting shared O-polysaccharides. If the effectiveness of the vaccine is reliant on these targeted polysaccharides, which are shared among all serovars within the same serogroup, this vaccine would offer cross protection against all Group B and Group D serovars, including the serovars with the next highest prevalence among hospitalized cases in Bamako, Mali (*S*. Stanleyville and *S*. Dublin, respectively) [13, 15–16].

Our findings have some limitations because of the lack of data on segments of the pediatric population where iNTS disease may be occurring but not detected with our surveillance. By focusing only on iNTS cases admitted to hospital, we did not model the overall burden of NTS in the Bamako pediatric population that would include children in the community with iNTS infections who were not ill enough for their caretakers to seek health care or who were brought to traditional healers. It also did not include children with severe iNTS disease who may not have had easy access to the hospital and thus may have died at home. Moreover, blood cultures and cultures of ordinarily sterile body fluids are not 100% sensitive in detecting invasive bacterial infections, particularly if antibiotics were administered prior to reaching the hospital. Thus, some iNTS cases may have been missed by our surveillance techniques, leading to an underestimation of the number of cases and the burden of NTS in the study population. However, cases hospitalized at HGT likely capture a substantial proportion of the more severe clinical forms of iNTS disease which likely have a higher case fatality than milder forms of iNTS disease. The impact of a NTS vaccine in preventing culture-negative severe iNTS cases could be estimated by noting the difference between the decrease in hospitalized iNTS cases following vaccine implementation and a decrease in all-cause hospitalizations [10]. Despite some knowledge gaps about the epidemiologic behavior of iNTS disease in the community, our model provides an informative view that should adequately assess the impact of introducing an effective vaccine.

The model presented herein is one of the first attempts to capture mathematically the epidemiologic dynamics of endemic pediatric iNTS disease in Africa and to predict the effects of future implementation of NTS vaccines currently in development on the disease burden. Several key features of the epidemiology of iNTS disease in Bamako, such as the force of infection, reservoirs of infection, and modes of transmission remain unknown and the specific effects of other factors known to modulate host risk and clinical disease severity such as HIV infection, malaria, and malnutrition [6, 7, 45] have not been formally measured. By relying on population data to inform the age-specific incidence of severe (hospitalized) iNTS disease, we indirectly captured some effects that subclinical infection and chronic carrier states may have without directly including them in the model, since such states have not yet been described for NTS cases [6]. Research efforts are underway in other venues to investigate risk factors, transmission modes, immunology, and natural reservoirs of iNTS.

Mali has a lauded EPI and thus immunization coverage in other venues may not be as high. However, the model can be used to predict outcomes with other location-specific estimates of NTS coverage. Our Markov Chain approach was well suited for incorporating the surveillance data representing the currently available numbers of hospitalized iNTS cases, but made the conservative assumption that infection pressure remained constant both in the natural history of the disease and in the face of vaccination. In future iterations we plan to amend and refine our model using data from other field investigations and use ordinary differential equation (ODE) and partial differential equation (PDE) models that include older children and adults to capture transmission dynamics and emergent properties of vaccination, such as herd immunity. As more literature is published on the epidemiology of iNTS disease, a more sophisticated computational model can be crafted that incorporates new data, adding further emphasis to the importance of implementing such a vaccine to protect young children against this invasive disease.

## Supporting Information

S1 Dataset Anonymized Clinical Dataset from HGT Surveillance. Includes anonymized identification number (ID), age groups as relative to the model (Age (months)), year admitted to the HGT (Year Admitted), *Salmonella* serovar (Serovar), and survival of the patient (Death; yes (Y) or no (N)).
(XLSX)
